# Clinical value of SIRT1 as a prognostic biomarker in esophageal squamous cell carcinoma, a systematic meta-analysis

**DOI:** 10.1515/med-2022-0454

**Published:** 2022-03-14

**Authors:** Yu-ling Zhang, Pei Chen, Ying Guo, Yan-jun Zhang

**Affiliations:** Department of Public Health, Jiangsu College of Nursing, Huai’an, Jiangsu Province, China; Department of Basic Medicine, Jiangsu College of Nursing, Qing Jiang pu District, Huai’an, Jiangsu Province, 223005, China; Department of Medical Laboratory, Huai’an Maternal and Child Health Hospital, Jiangsu Province, China

**Keywords:** SIRT1, ESCC, clinical features, progression, meta-analysis

## Abstract

Several studies reported that the expression of SIRT1 was associated with the clinical features of patients with esophageal squamous cell carcinoma (ESCC), but the function remains inconsistent. We conducted this study to illustrate the clinical value of SIRT1 expression in the early diagnosis and prediction of prognosis of ESCC. In this study, PubMed, Embase, and Web of Science were searched by two independent researchers and STATA14.0 software was used to conduct meta-analysis. The odds ratio with 95% confidence interval was used to estimate the pooled effect. Egger’s test and Begg’s funnel were used to assess publication bias. Sensitivity analysis was used to evaluate the reliability and stability of meta-analysis results. According to the inclusion and exclusion criteria, six studies were enrolled, including 811 cases of ESCC. Results of the meta-analysis indicated that SIRT1 was overexpressed in ESCC and the SIRT1 expression was closely related to the clinicopathological features of ESCC, such as tumor infiltration, tumor node metastasis (TNM) stage, and lymph node metastasis. In the survival analysis, high expression of SIRT1 represented a poor prognosis in ESCC patients. Our study demonstrated that SIRT1 was overexpressed in ESCC, and it might be a potential biomarker for progress of ESCC.

## 1 Introduction

Esophageal carcinoma is the eighth most common type of cancer worldwide and constitutes the sixth leading cause of cancer [1] accounting for over 600,000 new cases and 540,000 cancer deaths annually, which is 3.1% of all global new cancer cases and 5.5% of all cancer deaths [2]. The incident cases of esophageal carcinoma are expected to increase by roughly 35% from 2018 to 2030 worldwide and the estimated number of deaths are expected to increase by roughly 37% during this same timeframe [3]. In China, more than 90% of esophageal cancers are SCC (squamous cell carcinoma). Despite major advances in the fields of surgery, radiotherapy, and chemotherapy, the effectiveness of related treatments for ESCC (esophageal squamous cell carcinoma) is still very low. Due to its aggressive characteristics, the prognosis of ESCC is very poor, and the five-year survival rate is only 15–25% [4]. Early diagnosis of ESCC remains the best way to improve cure and survival rates [5]. Therefore, it is critical to explore effective biomarkers for early diagnosis of ESCC and predicting tumor progression and prognosis, which will significantly reduce mortality, especially in advanced or metastatic ESCC patients.

Sirtuins (SIRTs) are a conservative family of proteins. There are seven different subtypes (SIRT1–7), which mainly regulate the expression of multiple genes through acetylation of proteins and participate in the pathogenesis of many chronic diseases such as diabetes, cardiovascular diseases, and cancer. SIRT1 is a histone deacetylase and an important component of cell self-protection. It is located on the human chromosome 10q21.3 and is highly conserved, playing an important role in tissue and cell growth, aging, and apoptosis [6].

The mechanism of SIRT1 expression with the occurrence and progress of ESCC is still unclear. Several studies reported a relationship between SIRT1 and ESCC, but the results are inconsistent. He et al. [7], Zhang et al. [8], and Ma et al. [9] demonstrated that there was no significant difference of SIRT1 expression between infiltration (T1 + T2) and infiltration (T3 + T4) of ESCC. However, Yan et al. [10] and Chen et al. [11] reported that the SIRT1 expression was significantly lower in infiltration (T1 + T2) than that in infiltration (T3 + T4) of ESCC. Such inconsistent results were also observed in the TNM stage, lymph node metastasis, and other clinicopathological features of ESCC. Because of the inconsistent results of published studies and small sample size, the conclusion is unreliable according to a single clinical randomized controlled study. Therefore, we conducted this meta-analysis including all of the eligible studies to obtain a pooled effect to evaluate the association between SIRT1 expression and clinicopathological features of ESCC, providing basis for early diagnosis, treatment, and evaluation of progression and prognosis of ESCC.

## 2 Materials and methods

This meta-analysis was based on the preferred reporting items for systematic reviews and meta-analysis (PRISMA) guidelines [12].

### 2.1 Studies searching strategy

Published studies about SIRT1 expression and ESCC were searched in PubMed, Embase, and Web of Science by two independent researchers (up to July 2021). The following strategy and keywords were used for study searching, “SIRT1” or “Sirtuin1” and “Esophageal cancer” or “Esophageal tumor” or “Esophageal carcinoma”. Furthermore, “carcinoma” was replaced by “Esophageal squamous cell carcinoma” to identify any missing studies. The reference lists of retrieved studies were also manually reviewed to identify additional potentially relevant studies. No limitation on country, race, and language was added when studies were searched.

### 2.2 Inclusion and exclusion criteria

The included studies must meet the following criteria: (1) the studies explored the relationship between SIRT1 expression and ESCC, providing a clear detection and analysis method. (2) The studies provided the criteria for defining high and low expression of SIRT1 in ESCC tissues clearly. (3) Data of SIRT1 expression and clinicopathological features, such as differentiation, infiltration, and TNM clinical stage in ESCC can be obtained or calculated. (4) Repetitive data of articles published by the same research team in different journals, the largest sample size, or the latest published articles were included. The exclusion involved: (1) secondary research such as review, meta-analysis and case reports, and meeting papers. (2) Cell or animal research studies. (3) Studies with insufficient information or contradictions in data. (4) Duplicated studies.

### 2.3 Data extraction and quality assessment

#### 2.3.1 Data extraction

A prespecified, standardized data extraction form was used for data extracting, two researchers independently completed the data extraction and screening. Any disagreements were resolved by discussion to achieve a consensus. The extracted data mainly included the first author’s name, year of publication, number of cases and controls, testing method of SIRT1 expression, and clinicopathological features of ESCC.

#### 2.3.2 Variables

The clinicopathological features of ESCC included tumor size, differentiation, infiltration, TNM clinical stage, and lymph node metastasis. The tumor size of ESCC was grouped ≤5 cm and >5 cm. The tumor differentiation was grouped high, medium, and low in the included studies, and high and medium were combined into one group when the pooled effect was calculated. Tumor infiltration was grouped T1, T2, T3, and T4 in the included studies, and T1 and T2 were combined into one group and T3 and T4 to another group when the pooled effect was calculated. The clinical stage was grouped stage I, II, III, and IV in the included studies, and I and II were combined into one group and III and IV to another group when the pooled effect was calculated. The tumor lymph node metastasis was grouped as negative and positive. The HR of overall survival time was collected or infered through Kaplan-Meier curve.

#### 2.3.3 Quality assessment

The Newcastle-Ottawa Scale (NOS) was used to evaluate the quality of included studies. The scores ranged from 0 to 9, and the studies with 6 or more was regarded as high quality [13].

### 2.4 Statistical analysis

STATA 14.0 software was used to conduct statistical analysis. The relationship between SIRT1 expression and clinicopathological features or overall survival time of ESCC was conducted by pooled odds ratio (OR) or hazard ratio (HR) and 95% confidence interval (CI). The statistical significance of the OR or HR was analyzed by the *Z*-test and the corresponding *P* value. The heterogeneity test was performed by the *I* ^2^ test and the corresponding *P* value; when *I* ^2^ ≥ 50% and *P* ≤ 0.05, it meant there was significant heterogeneity among the included studies, and the random effects model was used; when *I* ^2^ < 50% and *P* > 0.05, it meant there was no significant heterogeneity among the included studies and the fixed effects model was used. Publication bias was tested through Egger’s test and Begg’s funnel. Sensitivity analysis was used to evaluate the reliability and stability of meta-analysis results. Two-tailed *P* ≤ 0.05 was regarded as statistically significant.

## 3 Results

### 3.1 Characteristics of the included studies

**Figure 1:**
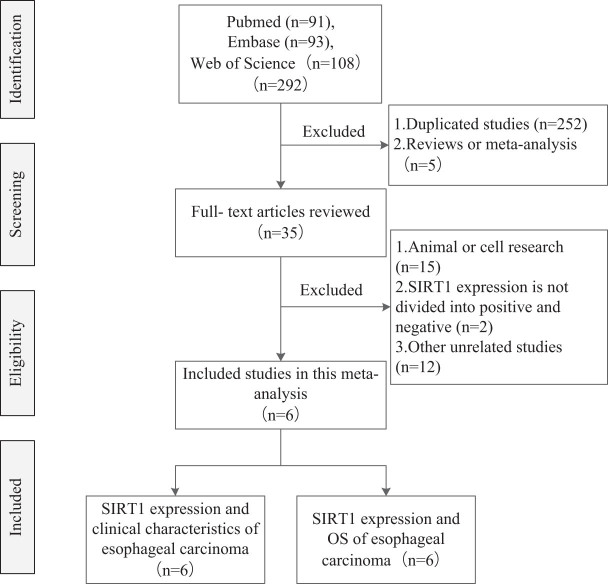
Flow diagram.

According to the inclusion and exclusion criteria, six studies were enrolled, including 811 cases of ESCC. Only one study reported SIRT1 expression in ESCC and control. Six studies reported the relationship between SIRT1 expression and differentiation in ESCC. Five studies reported the relationship between SIRT1 expression and infiltration in ESCC. Six studies reported the relationship between SIRT1 expression and the TNM clinical stage in ESCC. Four studies reported the relationship between SIRT1 expression and lymph node metastasis in ESCC. Six studies reported the relationship between SIRT1 expression and the overall survival time in ESCC. Six studies reported the relationship between SIRT1 and tumor size, age, and gender in ESCC (Table 1). All the studies inspected SIRT1 expression with immunohistochemistry. The flow diagram of study searching and screening is shown in Figure 1.

**Table 1:** Characteristics of included studies

Study	Year	Case	Control	Clinicopathological features	NOS score
He et al. [7]	2015	86	—	1, 2, 3, 4, 5, 6, 7, 8	6
Zhang et al. [8]	2013	176	32	1, 2, 3, 4, 5, 6, 7, 8	8
Ma et al. [9]	2018	155	—	1, 2, 4, 5, 6, 7, 8	7
Yan et al. [10]	2020	93	—	1, 2, 3, 4, 5, 6, 7, 8	6
Chen et al. [11]	2014	206	—	1, 2, 3, 4, 5, 6, 8	7
Han et al. [14]	2018	95	—	1, 2, 3, 4, 6, 8	6

1, Age; 2, gender; 3, tumor size; 4, differentiation; 5, infiltration; 6, TNM stage; and 7, lymph node metastasis; 8, overall survival time.

### 3.2 The expression of SIRT1 in ESCC

**Figure 2:**
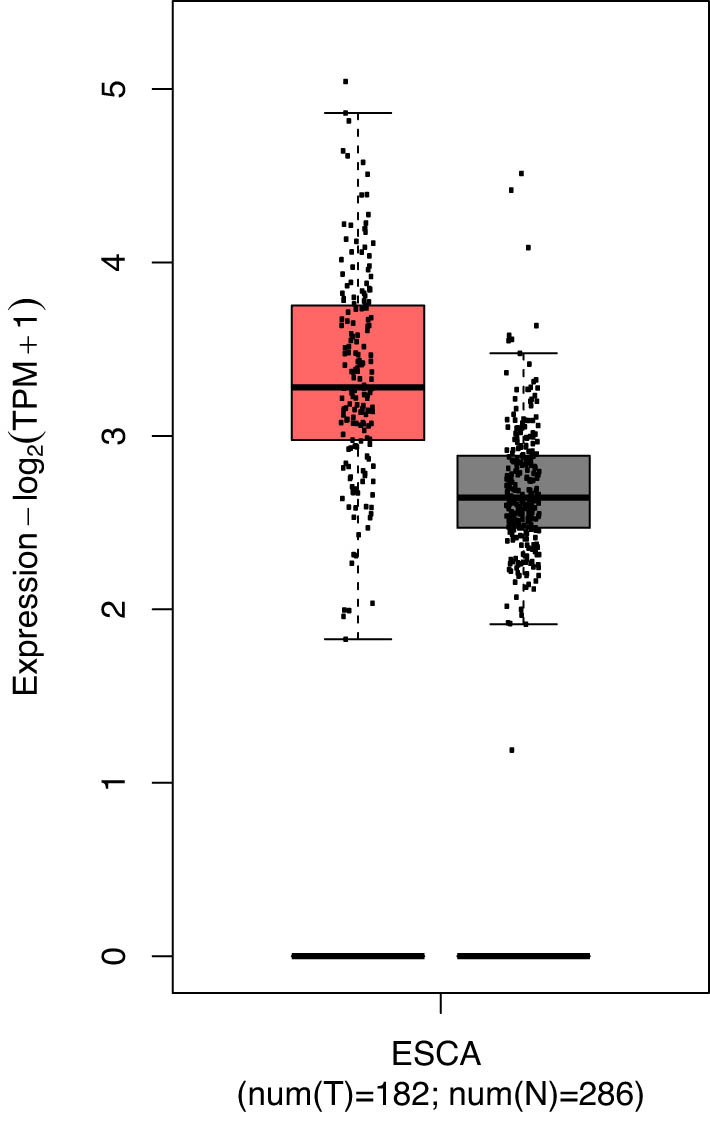
SIRT1 expression in ESCC and normal tissues in the TCGA database.

Because only one study was enrolled about the expression of SIRT1 in ESCC and control, meta-analysis could not be conducted. The result of this study indicated that SIRT1 was overexpressed in ESCC patients than in the control, which was consistent with the data in The Cancer Genome Atlas (TCGA) database (http://gepia2.cancer-pku.cn) (Figure 2).

### 3.3 Relationship between SIRT1 expression and clinicopathological features of ESCC

#### 3.3.1 Relationship between SIRT1 expression and differentiation of ESCC

**Figure 3:**
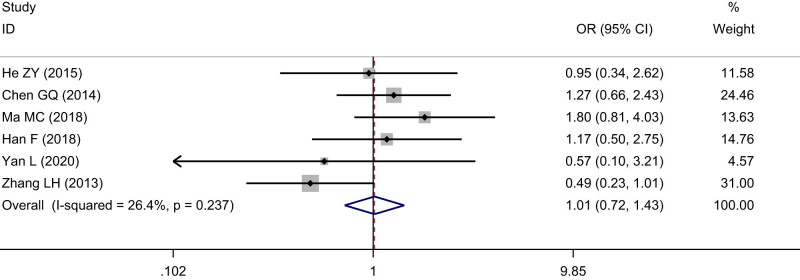
Forest figure of relationship between the expression of SIRT1 and differentiation of ESCC.

Six studies including 776 cases were enrolled about SIRT1 expression and differentiation of ESCC. The cases with positive SIRT1 expression were 181 among 360 cases in medium and high differentiation of ESCC, with a positive rate of 50.28%. The cases with positive SIRT1 expression were 246 among 416 cases in low differentiation of ESCC, with a positive rate of 59.13%. The heterogeneity test indicated that no significant heterogeneity was observed among the included studies (*I* ^2^ = 26.4%, *P* = 0.24), and the fixed effects model was used to calculate the pooled effect variable. The SIRT1 expression was a little lower in medium and high differentiation than that in low differentiation of ESCC (OR = 1.01, 95% CI: 0.72–1.43), but the difference was not statistically significant (*Z* = 0.09, *P* = 0.93), (Figure 3). No significant publication bias was observed (*t* = 1.73, *P* = 0.16).

#### 3.3.2 Relationship between SIRT1 expression and infiltration of ESCC

**Figure 4:**
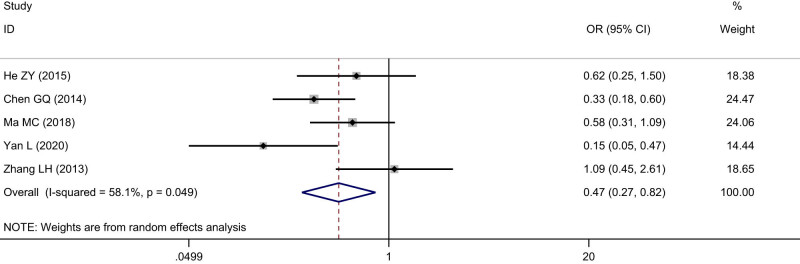
Forest figure of relationship between the expression of SIRT1 and infiltration of ESCC.

Five studies including 716 cases were enrolled about SIRT1 expression and infiltration of ESCC. The cases with positive SIRT1 expression were 130 among 301 cases in tumor infiltration (T1 + T2) of ESCC, with a positive rate of 43.19%. The cases with positive SIRT1 expression were 252 among 415 cases in tumor infiltration (T3 + T4) of ESCC, with a positive rate of 60.72%. The heterogeneity test indicated that heterogeneity was observed among the included studies (*I* ^2^ = 58.1%, *P* = 0.05), and the random effects model was used to calculate the pooled effect variable. The SIRT1 expression was significantly lower in infiltration (T1 + T2) than that in infiltration (T3 + T4) of ESCC (OR = 0.47, 95% CI: 0.27–0.82), the difference was statistically significant (*Z* = 2.67, *P* < 0.05), (Figure 4). No significant publication bias was observed (*t* = 0.51, *P* = 0.66).

#### 3.3.3 Relationship between SIRT1 expression and the TNM stage of ESCC

**Figure 5:**
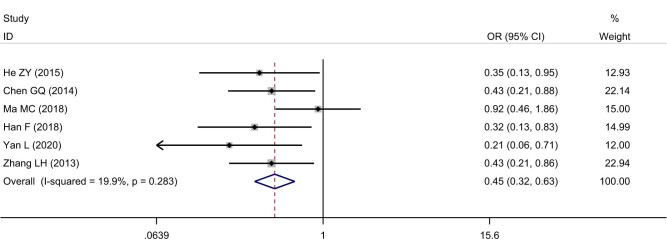
Forest figure of relationship between the expression of SIRT1 and the TNM stage of ESCC.

Six studies including 811 cases were enrolled about SIRT1 expression and the TNM stage of ESCC. The cases with positive SIRT1 expression were 176 among 359 cases in the tumor stage (I + II) of ESCC, with a positive rate of 49.03%. The cases with positive SIRT1 expression were 269 among 452 cases in the tumor stage (III + IV) of ESCC, with a positive rate of 59.51%. The heterogeneity test indicated that no significant heterogeneity was observed among the included studies (*I* ^2^ = 19.9%, *P* = 0.28), and the fixed effects model was used to calculate the pooled effect variable. The SIRT1 expression was lower in the tumor stage (I + II) than that in stage (III + IV) of ESCC (OR = 0.45, 95% CI: 0.32–0.63), and the difference was statistically significant (*Z* = 4.72, *P* < 0.05), (Figure 5). No significant publication bias was observed (*t* = 2.42, *P* = 0.07).

#### 3.3.4 Relationship between SIRT1 expression and lymph node metastasis of ESCC

**Figure 6:**
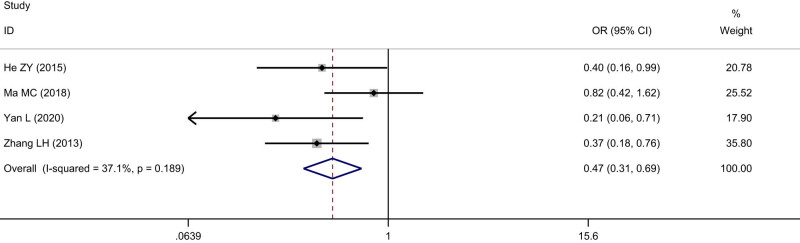
Forest figure of relationship between the expression of SIRT1 and lymph node metastasis of ESCC.

Four studies including 510 cases were enrolled about SIRT1 expression and lymph node metastasis of ESCC. The cases with positive SIRT1 expression were 117 among 239 cases without tumor lymph node metastasis of ESCC, with a positive rate of 48.95%, The cases with positive SIRT1 expression were 170 among 271 cases with tumor lymph node metastasis of ESCC, with a positive rate of 62.73%. The heterogeneity test indicated that heterogeneity was observed among the included studies (*I* ^2^ = 37.1%, *P* = 0.19), and the fixed effects model was used to calculate the pooled effect variable. The result indicated that SIRT1 expression was lower in ESCC without lymph node metastasis than that with tumor lymph node metastasis (OR = 0.47, 95% CI: 0.31–0.69), and the difference was statistically significant (*Z* = 3.76, *P* < 0.05) (Figure 6). No significant publication bias was observed (*t* = 2.10, *P* = 0.17).

#### 3.3.5 Relationship between SIRT1 expression and tumor size, age, and gender of ESCC

Five studies including 656 cases reported relationship between SIRT1 expression and tumor size in ESCC. No significant difference of SIRT1 expression was observed between tumor size (≤5 cm) and tumor size (>5 cm) (OR = 1.14, 95% CI: 0.78–1.66). Six studies including 811 cases reported relationship between SIRT1 expression and age and gender in ESCC. No significant difference of SIRT1 expression was observed between age (≤60 cm) and age (>60 cm) (OR = 1.24, 95% CI: 0.92–1.67) and was also observed in males and females (OR = 1.07, 95% CI: 0.74–1.54) (Table 2).

**Table 2:** Meta-analysis of SIRT1 expression and tumor size, age, and gender of ESCC

Variables	Included studies	SIRT1 expression			Heterogeneity		Publication bias	
		OR (95% CI)	*Z*	*P*	*I* ^2^	*P*	*t*	*P*
Tumor size	5	1.14 (0.78–1.66)	0.67	0.50	0.0%	0.81	3.78	0.03
Age	6	1.24 (0.92–1.67)	1.39	0.16	0.0%	0.99	4.54	0.01
Gender	6	1.07 (0.74–1.54)	0.35	0.72	0.0%	0.42	1.40	0.23

#### 3.3.6 Relationship between SIRT1 and overall survival of ESCC

**Figure 7:**
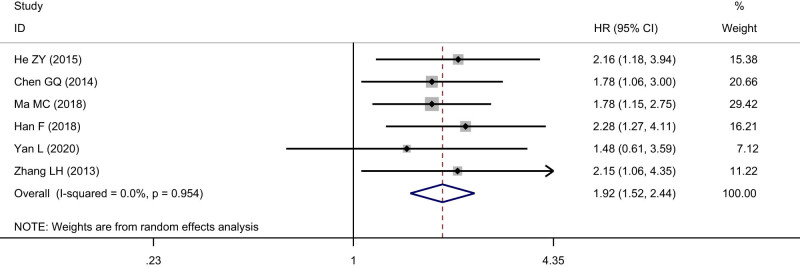
Relationship between SIRT1 and overall survival of ESCC.

Six studies including 811 cases reported the relationship between the overexpression of SIRT1 and the overall survival time of ESCC patients after surgery. The result indicated that the overall survival time of ESCC patients with positive SIRT1 expression was significantly lower than that in patients with negative SIRT1 expression after surgery (HR = 1.92, 95% CI: 1.52–2.44), and the difference was statistically significant (*Z* = 5.43, *P* < 0.05) (Figure 7), suggesting that SIRT1 is closely related to the prognosis of ESCC. The heterogeneity test indicated that no heterogeneity was observed among the included studies (*I* ^2^ = 0.0%, *P* = 0.95). No publication bias was observed (*t* = 2.11, *P* = 0.10).

### 3.4 Publication bias and sensitivity analysis

Publication bias was tested through Egger’s test and Begg’s funnel. No significant publication bias was observed in the analysis of SIRT1 expression and differentiation, infiltration, TNM stage, lymph node metastasis, and the overall survival time in ESCC (*P* > 0.05). But some publication bias was observed in the analysis of SIRT1 expression and tumor size and age in ESCC (*P* < 0.05).

The results of sensitivity analysis indicated that our meta-analysis was stable.

## 4 Discussion

SIRT1 is one of the SIRTs, which participates in a large number of biological processes including DNA repair, apoptosis and inflammation [15], aging [16], and autophagy. It plays a crucial role in protection against various human diseases, including metabolic syndromes, cardiovascular diseases, and tumorigenesis. A lot of studies indicated that SIRT1 could serve as a candidate biomarker of human cancer. However, as a crucial regulator, the function of SIRT1 in ESCC has not been well understood.

Our study demonstrated that SIRT1 overexpressed in ESCC compared with normal control. To explore the status of SIRT1 expression in the progress and prognosis of ESCC, we analyzed the relationship between SIRT1 and differentiation, infiltration, TNM stage, lymph node metastasis, and the overall survival time of ESCC. According to the results of our meta-analysis, the pooled effect indicated that SIRT1 overexpressed in tumor infiltration (T3 + T4), TNM stage (III + IV), and positive cases lymph node metastasis of ESCC (*P* < 0.05). No significant correlation was found between SIRT1 expression and tumor differentiation, tumor size, age, and gender in cases of ESCC (*P* > 0.05). Our result also indicated that the overall survival time of ESCC patients with positive SIRT1 expression was significantly lower than that of patients with negative SIRT1 expression after surgery (HR = 1.92), which meant that SIRT1 might be a cancer promoting factor. In our meta-analysis, heterogeneity among the included studies was also analyzed, and the sample size and study quality might be the main sources of heterogeneity.

Our study confirmed that the expression of SIRT1 was closely related to ESCC, and the expression of SIRT1 might be a potential biomarker to identify the progress and prognosis of ESCC. At present, the mechanisms of SIRT1 in tumorigenesis and development are still unclear. The mechanisms are mainly as follows: (1) SIRT1 effects metabolism of tumor cells. The activity of SIRT1 is often coupled with homeostasis and metabolism. Chen et al. [17] reported that SIRT1 promoted GLUT1 expression and progression in bladder cancer via regulation of glucose uptake. Simmons et al. [18] found that SIRT1 influenced pathways that provided an alternative means of deriving energy (such as fatty acid oxidation and gluconeogenesis) when a cell encountered nutritive stress and could therefore lead to altered lipid metabolism in various pathophysiological contexts. The survival function of SIRT1 may reflect abnormal cancer metabolism and identifies SIRT1 as a target for anticancer therapy. (2) SIRT1 engenders an error in repairing damaged DNA. SIRT1 can interact with distinct proteins from the main DNA repair mechanisms and DNA damage response (DDR) pathways recruiting them to DNA damage foci or activating the proteins involved in DNA repair by deacetylating them. These processes help the cells to live without damaged DNA but also prone to errors, leading to mutations and abnormal epigenetic marks [19]. The relationship between the function of SIRT1 on DNA repairment and tumorigenesis is not fully understood. Studies to elucidate these pathways will provide a breakthrough in cancer biology. (3) SIRT1 plays a role in tumor promoting by affecting cell proliferation, metastasis, and apoptosis. Garten et al. [20] reported that overexpression of SIRT1 significantly decreased sorafenib-induced apoptosis, which could be an underlying mechanism of resistance to sorafenib treatment in hepatocellular carcinoma (HCC). Zhang et al. [21] found that SIRT1 functioned as a tumor suppressor encouraging gastric cancer progression through the activation of STAT3/MMP-13 signaling, inhibited proliferation, and metastasis of gastric cancer. The function of promoting the proliferation and metastasis of SIRT1 was also observed in pancreatic cancer [22], colorectal cancer [23], lung cancer [24], and other cancers. Effects on immune responses [25], autophagy [26,27], and inflammation [28] were also observed in published studies to illustrate the mechanism of SIRT1 on cancer cell proliferation, metastasis, and apoptosis.

Although efforts had been made, our studies still had some limitations. (1) Due to the limitations of published studies, the population included in this study is mainly the Han Chinese population, and some bias might exist in the analysis. (2) Due to limitation of included studies, the sample size was relatively small. (3) Due to different criteria for judging the positive or negative expression of SIRT1, the included studies had some heterogeneity. (4) Since the included studies were all case-control studies, it was difficult to determine whether SIRT1 was the cause or result of ESCC. (5) Because some included articles did not report detailed survival data, we could only use the Kaplan–Meier curve in the survival analysis to infer the corresponding results, which might overestimate or underestimate the real survival data. Hence, our results of this meta-analysis should be verified by additional larger sample size and well-designed clinical randomized controlled studies in different races, and larger sample size cohort study will also be conducted to verify whether SIRT1 overexpression was the cause or result of ESCC.

## 5 Conclusion

We confirmed that SIRT1 was overexpressed in ESCC, and the expression of SIRT1 was closely related to the invasion, metastasis, and prognosis of ESCC. SIRT1 might be a potential biomarker to identify the progress and prognosis in ESCC.
